# Simultaneous gene expression profiling in human macrophages infected with *Leishmania major *parasites using SAGE

**DOI:** 10.1186/1471-2164-9-238

**Published:** 2008-05-21

**Authors:** Fatma Z Guerfali, Dhafer Laouini, Lamia Guizani-Tabbane, Florence Ottones, Khadija Ben-Aissa, Alia Benkahla, Laurent Manchon, David Piquemal, Sondos Smandi, Ons Mghirbi, Thérèse Commes, Jacques Marti, Koussay Dellagi

**Affiliations:** 1 Laboratoire d'Immuno-Pathologie, Vaccinologie et Génétique Moléculaire (LIVGM), WHO Collaborating Center for Research and Training in Leishmaniasis, Institut Pasteur de Tunis, 13 place Pasteur, BP 74, 1002 Tunis-Belvédère, Tunisia; 2 Laboratoire International Associé (LIA) "Ingénierie Biomoléculaire", Centre National de Recherche Scientifique (CNRS), France; 3 Groupe d'Etude des Transcriptomes (GET), Institut de Génétique Humaine, UPR CNRS 1142, 141, rue de la Cardonille, Montpellier cedex5, 34396, France; 4 Skuld-Tech, 88, cour des Camisards, Montpellier, 34080, France

## Abstract

**Background:**

*Leishmania *(*L*) are intracellular protozoan parasites that are able to survive and replicate within the harsh and potentially hostile phagolysosomal environment of mammalian mononuclear phagocytes. A complex interplay then takes place between the macrophage (MΦ) striving to eliminate the pathogen and the parasite struggling for its own survival.

To investigate this host-parasite conflict at the transcriptional level, in the context of monocyte-derived human MΦs (MDM) infection by *L. major *metacyclic promastigotes, the quantitative technique of serial analysis of gene expression (SAGE) was used.

**Results:**

After extracting mRNA from resting human MΦs, *Leishmania*-infected human MΦs and *L. major *parasites, three SAGE libraries were constructed and sequenced generating up to 28,173; 57,514 and 33,906 tags respectively (corresponding to 12,946; 23,442 and 9,530 unique tags). Using computational data analysis and direct comparison to 357,888 publicly available experimental human tags, the parasite and the host cell transcriptomes were then simultaneously characterized from the mixed cellular extract, confidently discriminating host from parasite transcripts. This procedure led us to reliably assign 3,814 tags to MΦs' and 3,666 tags to *L. major *parasites transcripts. We focused on these, showing significant changes in their expression that are likely to be relevant to the pathogenesis of parasite infection: (i) human MΦs genes, belonging to key immune response proteins (e.g., IFNγ pathway, S100 and chemokine families) and (ii) a group of *Leishmania *genes showing a preferential expression at the parasite's intra-cellular developing stage.

**Conclusion:**

Dual SAGE transcriptome analysis provided a useful, powerful and accurate approach to discriminating genes of human or parasitic origin in *Leishmania*-infected human MΦs. The findings presented in this work suggest that the *Leishmania *parasite modulates key transcripts in human MΦs that may be beneficial for its establishment and survival. Furthermore, these results provide an overview of gene expression at two developmental stages of the parasite, namely metacyclic promastigotes and intracellular amastigotes and indicate a broad difference between their transcriptomic profiles. Finally, our reported set of expressed genes will be useful in future rounds of data mining and gene annotation.

## Background

Co-evolution of humans and pathogens has exerted a dual selective pressure on the immune system of the host that strives to control infection and on the pathogens, which have developed various strategies to circumvent the host's immune responses.

*Leishmania *(*L*) parasites are obligate intracellular pathogens that preferentially invade macrophages (MΦs) where they replicate, ultimately causing a heterogeneous group of diseases that affects millions of people mainly in subarid, tropical and subtropical areas [1]. In view of their wide distribution, leishmaniasis remain embedded in impoverished populations and represent a paradigm of neglected diseases [2].

To establish infection, the flagellated metacyclic promastigotes must enter MΦs and avoid triggering host responses. Since MΦs play a dual function in infection, acting as a safe shelter for parasites but also as their ultimate killer, these cells are the alpha and the omega for host resistance or susceptibility to *Leishmania *infection. Cellular events occurring early during MΦ-parasite interactions are likely to influence the fate of infection. MΦs are able to secrete a remarkably diverse set of regulators known to influence the physiological functions and differentiation of neighboring cells. Thus, activation of the innate immunity is required, by migrating parasitized dendritic cells to trigger an adaptive immune response of the Th1-type. The latter induces interferon (IFN) γ-activated MΦs to kill *Leishmania *parasites, promote disease healing and regulate resistance to re-infection as well as vaccine-induced immunity [3].

*Leishmania *have developed a range of sophisticated mechanisms to subvert the leishmanicidal activities of MΦs, by altering gene expression for cytokines, chemokines, transcription factors, membrane receptors and molecules involved in signal transduction in infected cells [4,5]. Although a wealth of crucial information has already been reported on the matter, it generated only a segmented view that hardly recognizes the full value of the biological consequences of this host-parasite conflict on a more global scale.

There is obviously a need for a high-throughput approach that generates a global view, in order to identify the salient modifications of the biological pathways triggered by intracellular parasitism. Applying transcriptomics to study host-pathogen interactions has already contributed important insights to the understanding of the mechanisms of pathogenesis, and it is expanding further with the accumulation of genomic sequences of host organisms (e.g., human) and their pathogens [6]. Indeed, several studies analyzing the human MΦ transcriptome upon viral [7], bacterial [8] or fungal [9] infections have been published. However, to our knowledge, only one study, using the microarray technique has described the effect of *L. major *infection on the transcriptome of human MΦs [10]. More recently, a paper has described at the global scale the abrogation in the human monocytic THP1 cell line of IFNγ gene expression by this parasite species [11].

Gene expression profiling has also been used in several studies of different pathogenic microorganisms, including protozoan parasites [12]. These studies were applied to *L. major, L. donovani*, *L. infantum *and *L. mexicana *spp., using differential display or array probes amplified from either cDNA or randomly sheared genomic DNA; these techniques identified differentially expressed genes at different developmental stages [13-20].

Compared to other transcriptomic methods, Serial Analysis of Gene Expression (SAGE) technology has proved to be a powerful tool for the quantitative cataloguing and comparison of genes expressed in cells or tissues from various physiological and pathological conditions. Additionally, SAGE allows one to study the expression profiles of both known and unknown genes and as a result contributes to better genome annotation [21]. This technology was successfully applied to study the transcriptome of different parasites e.g., *Plasmodium falciparum *[22]. *Schistosoma mansoni *[23] and *Trypanosoma congolense *[24], among others [21].

As far as we know, this is the first study using the SAGE strategy that provides a high-throughput simultaneous analysis of gene expression in the context of the *Leishmania*-human MΦ encounter. Although the impact of the parasite on the human transcriptome appeared globally marginal, we identified several genes corresponding to diverse functional pathways that were differentially expressed upon infection, suggesting their likely involvement in the infectious process. Interestingly, we individualized genes involved in complement or IFNγ pathways, and others belonging to S100 proteins, MHC molecules, apoptosis, cytokines and chemokines families. Concurrently, our SAGE analysis unveiled a deep variation in parasite transcript abundance; such characterized transcripts could contribute to understanding the dynamics of gene expression in the intracellular parasite-stage.

## Results

Three SAGE libraries were generated from: (i) resting human MΦs (monocyte-derived MΦs or MDMs), (ii) human MΦs infected with *L. major *metacyclic promastigotes ("MDM+Lm", infection rates ranged from 87% to 92%) and (iii) *L. major *metacyclic promastigotes (Lm). A total of 28,173, 57,514 and 33,906 tags (corresponding to 12,946, 23,442 and 9,530 unique tags) were obtained from each library respectively (Table 1). Additional file 1 (Unique SAGE tags as a function of total sequenced tags in the different constructed libraries) shows the unique SAGE tags as function of total sequenced tags (for all tags panel A and for tags present at least twice, panel B). The entire dataset discussed in this publication has been deposited in NCBI Gene Expression Omnibus [25] and is accessible through GEO series accession number [GSE10442].

**Table 1:** Distribution of sequenced tags from different SAGE libraries.

	**Libraries**		
	**MDM**	**MDM+Lm**	**Lm**

**No of tags**	28,173	57,514	33,906
	(12,946)	(23,442)	(9,530)
**1 copy**	10,494	17,172	6,564
	(10,494)	(17,172)	(6,564)
**2 copies**	2,206	5,512	2,746
	(1,103)	(2,756)	(1,373)
**3 copies**	1,233	3,549	1,668
	(411)	(1,183)	(556)
**4 – 10 copies**	3,906	10,019	5,113
	(686)	(1,794)	(901)
**11 – 20 copies**	1,833	4,020	2,163
	(127)	(284)	(149)
**21 – 100 copies**	4,432	8,463	6,149
	(107)	(211)	(291)
**> 100 copies**	4,069	8,774	9,700
	(18)	(42)	(42)
**Occurrence up to**	689	720	753

This table shows number of tags present at 1, 2, 3, 4 to 10, 11 to 20, 21 to 100 and > 100 copies and number of occurrences in each library. Numbers in parentheses indicate different unique tags in each category.

### Data analysis allows good discrimination between human and parasite tags generated in the same "MDM+Lm" mixed SAGE library

**Figure 1:**
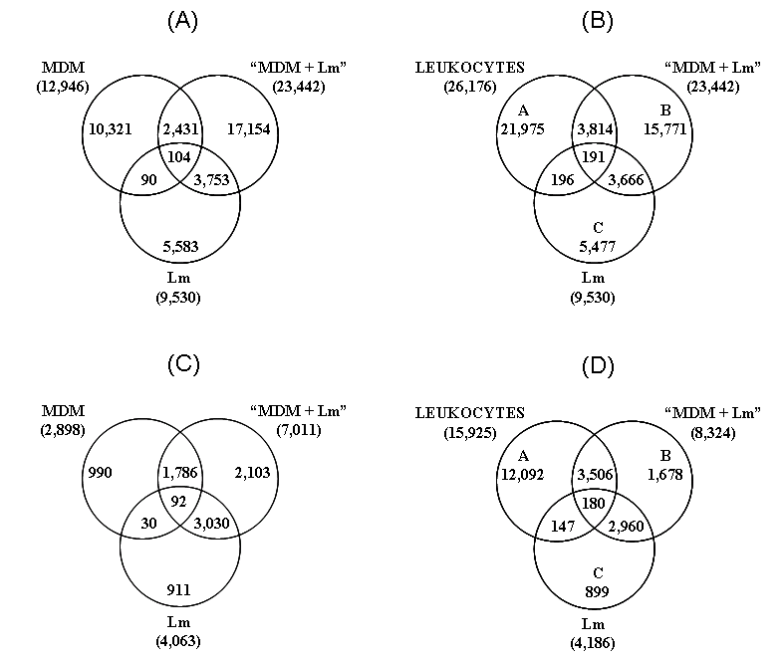
**Venn diagram comparing the parasite-infected MDM and *L. major *SAGE libraries with the MDM non-infected library or with other publicly available leukocyte libraries**. This figure is not drawn to scale. All unique tags species detected within the "MDM+Lm" library were compared to unique tags from the Lm and MDM libraries (A), or from the Lm library and a collection of publicly available human leukocyte tags (B). Panels C and D show comparisons after withdrawing tags found only once (unless present twice in another library).

To identify the transcripts that were modulated upon infection, we compared the three libraries that were constructed. We found that the MDM and Lm libraries had 194 tags in common and 3,857 tags were shared by the "MDM+Lm" and Lm libraries. In addition, 2,535 tags were common between MDM and "MDM+Lm" libraries (Figure 1A). Unexpectedly, this initial analysis showed that a large number of tags were specifically present in "MDM+Lm" library.

In order to more confidently assign these tags to a human origin, they were compared to an assembled composite matrix containing a total of 26,176 unique tags and built from (i) nine publicly available leukocyte SAGE libraries, that were generated from freshly isolated monocytes, M-CSF differentiated, GM-CSF differentiated and LPS activated cells, immature and mature monocyte-derived dendritic cells and un-fractionated populations of leukocytes, (ii) the non-infected MDM library (this paper) and (iii) a second in-house-generated MDM-M library (Ottones et al., unpublished data; see Methods section for details).

As shown on the Venn diagram in Figure 1B, only 191 tags were shared by all libraries (ABC subset) and 196 tags (AC subset) were shared by the Lm library (referred to as C) and the non-infected human leukocyte libraries (referred to as A). This low level (1.4%) of synonymy between human and parasite tags indicated that we could accurately discriminate human transcripts from parasite transcripts in the infected "MDM+Lm" sample (referred to as B). It is not excluded that some of the 3,814 human tags sorted in AB might correspond to parasite tags specifically expressed by the amastigote stage and absent in the promastigote-derived library. However, in this case we assumed that their number was likely to be in the same range as in AC and ABC, so that most AB tags could be reasonably considered as MΦ-specific.

It must be stressed here that the apparently large number of "MDM+Lm"-specific tags must be interpreted with caution because most of them were observed only once and may result from sequencing errors, the major source of noise inflating the number of unique tags. Indeed, when we reanalyzed the data excluding tags that appeared only once (unless present at least twice in another library), we ended up with 3,506 human tags sorted in AB, 2,960 tags common to the Lm and "MDM+Lm" libraries (BC subset, Figure 1D). Interestingly, when excluding unique tags, number of these present only in "MDM+Lm" library dropped from 15,771 to 1,678 tags. In fact, most of the tags occurring at high frequencies in the "MDM+Lm" library were sorted into the AB or BC subsets. Since these tags could be safely considered as identifying human (AB) or parasite (BC) transcripts, their respective frequencies could be taken as representative of the actual figure of the two species-specific transcripts in the initial mRNA inputs. Thus, we estimated that human and parasite mRNAs contributed to 51% and 49% (54% and 46% for tags > 1), respectively, of the "MDM+Lm" sample.

### Impact of *L. major *infection on MΦ transcriptome

**Figure 2:**
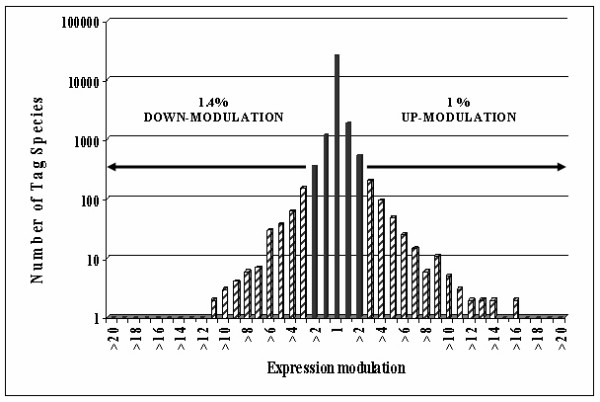
**Comparison of gene expression modulation in the *L. major*-infected "MDM+Lm" library to MDM library**. A semi-logarithmic plot shows that both up- and down-modulated tags were decreased within a bell-shaped curve except for a tail corresponding to tags upregulated 12 to 16 times. The relative expression of each transcript was determined by dividing the number of tags observed in the MDM library by the number of the same tags observed in the "MDM+Lm" library. To avoid division by 0, we used a tag value of 1 for any tag that was not detectable. These ratios are plotted on the abscissa. The number of tag species comprising each ratio is plotted on the ordinate.

To investigate MΦ tags that were modulated by *Leishmania *infection, we compared total tags present in the MDM library to those present in the "MDM+Lm" library, after withdrawing tags of parasitic origin. Most tags were expressed at similar levels between resting and *Leishmania*-infected MDM. A semi-logarithmic plot (Figure 2) showed that both up- and down-modulated tags were distributed within a bell-shaped symmetric curve, though tailed for the tags up-regulated 12–16 times. This ratio profile clearly indicates that only some transcripts (1.4% down-modulated, 1% up-modulated) were altered by *Leishmania *infection. A scatter plot showing statistically scaled modulated transcripts is shown in Additional file 2: Scatter plot showing the comparison of MDM versus "MDM+Lm" SAGE libraries.

Starting with the matrix registering initial SAGE data, we recalculated tag frequencies in each of the 11 libraries, replaced by the nearest integer of tag frequencies for 10,000 counts. For every tag, the sum of normalized frequencies was calculated and tags were discarded for values less than 2. The resulting matrix (2,918 rows) was split into two parts: the first registering the 500 tags with the highest sum of frequencies (Top500) and the second registering tags with lower frequencies (2,418).

**Figure 3:**
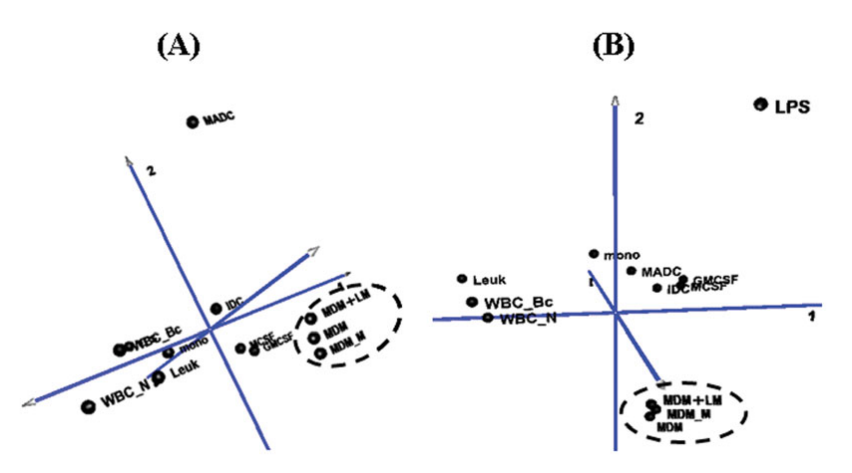
**Covariance Analysis of Gene expression profiles raised with the 500 most abundant tags (A) or with the next 2418 tags (B)**. This figure shows similarities of transcriptome profiles between different SAGE tag collections. "MDM+Lm" corresponds to MDM-specific genes extracted from the *Leishmania*-infected sample, MDM to the library built with the same MDM preparation and MDM-M to a second in-house library prepared independently with MDMs raised in similar conditions from another pool of donors (Ottones et al., unpublished data). Mono, LPS, GM-CSF, M-CSF, IDC, MADC, leuk, WBC-N and WBC-Bc correspond to publicly available SAGE libraries (see Methods' section).

Using Principal Component Analysis, observable either on 2D (not illustrated) or 3D graphs (Figure 3), landscapes generated with the Top500 dataset showed that the closest relationship was between "MDM+Lm" and their MDM control, and both were in the vicinity of the MDM-M sample. For the 2,418 dataset, the closest relationship was between the MDM and MDM-M libraries. Data analysis by Hierarchical Clustering using various modules of the TIGR MultiExperiment Viewer Package (MeV 4.0, 2006) led to similar conclusions (Additional file 3: Hierarchical Clustering raised with the 500 most abundant tags or with the next 2,418 tags).

Taking these data sets as a whole, MDM infected with *L. major *parasites showed a transcriptional profile closer to that of non-infected cells but clearly different from that of LPS-activated MΦs. In addition, the expression profiles of MDM, whether infected or not, were the closest to those of GM-CSF- and M-CSF-elicited cells.

These results globally indicate that the internalization of viable *Leishmania *parasites in macrophage and their intracellular multiplication appear to induce only minor changes in the basal transcriptome profile with no indication of an obvious inflammatory response. Nevertheless, a detailed comparison of MDM and "MDM+Lm" profiles revealed changes that might be biologically relevant to the infectious process.

### Quantitative PCR experiments confirmed the changes in gene expression detected in human SAGE libraries

Human tags were assigned to their corresponding genes using Preditag^® ^software [26] and BLAST and then to their related biological processes using Gene Ontology [27]. Quantitative real-time PCR was then used to assess the accuracy of the generated data. Several candidate families of genes showing differential expression patterns in our human SAGE libraries were selected. To compare the Q-PCR and SAGE data, "MDM+Lm"/MDM, expression ratios were calculated (Table 2).

**Table 2:** Quantitative RT-PCR validation of human gene transcripts.

*Gene symbol*	*Gene name*	*Fold Change*	
		**MDM+Lm/MDM Ratio&occurrences**	**2^-ΔΔCT^**

**Chemokine activity**			

*IL-8*	Interleukin 8	7.5 (15:2)	20
*CXCL3*	Chemokine (C-X-C motif) ligand 3	8 (8:1)	11.67
*CXCL9*	Chemokine (C-X-C motif) ligand 9	0.25 (2:8)	0.19
*CXCL10*	Chemokine (C-X-C motif) ligand 10	0.25 (0:4)	0.15

**Cytoskeleton**			

*GSN*	Gelsolin (amyloidosis, Finnish type)	0.44 (12:27)	0.29
*ADFP*	Adipose differentiation-related protein	13 (13:1)	2.43
*ACTG1*	Actin, gamma 1	0.62 (5:8)	0.40

**Extra-cellular space**			

*MMP12*	Matrix metallopeptidase 12 (macrophage elastase)	0.46 (2:9)	0.22
*PTPNS1*	Protein tyrosine phosphatase, non-receptor type S1	0.12 (1:8)	0.57
*FN1*	Fibronectin 1	0.05 (1:19)	0.28

**Inflammatory Response**			

*FCGR3*	Fc fragment of IgG, low affinity IIIb, receptor (CD16)	0.22 (2:9)	0.22
*C5R1*	complement component 5 receptor 1 (C5a ligand)	0.21 (3:14)	0.65
*CHIT1*	chitinase 1 (chitotriosidase)	0.33 (0:3)	0.86
*TNFSF10*	tumor necrosis factor (ligand) superfamily, member 10	**8 (8:1)**	**0.66**

**Membrane-associated protein**			

*AP2S1*	Adaptor-related protein complex 2, sigma 1 subunit	0.50 (1:2)	0.38
*GPNMB*	Glycoprotein (transmembrane) nmb	**0.81 (36:44**	**1.07**
*CD164*	CD164 molecule, sialomucin	0.16 (1:6)	0.79
*SCARB2*	Scavenger receptor class B, member 2	0.25 (0:4)	0.66

**Processing, folding and targeting**			

*SRP9*	signal recognition particle	0.16 (1:6)	0.49
*RPL13A*	Ribosomal protein L13a	0.44 (12:27)	0.28
*LGMN*	Legumain	**7 (7:1)**	**0.90**
*HSPA8*	Heat shock 70 kDa protein 8	0,33 (5:15)	0.58

**Response to stress**			

*SOD2*	Superoxide dismutase 2, mitochondrial	2.5 (15:6)	2.17
*GLRX*	Glutaredoxin (thioltransferase)	0.66 (4:6)	0.78
*MX1*	Myxovirus resistance1, IFN-inducible protein p78	3 (9:3)	2.21
*P4HB*	Procollagen-proline, 2-oxoglutarate 4-dioxygenase	0.27 (3:11)	0.63

**Signal transduction events**			

*S100A8*	S100 calcium binding protein A8 (calgranulin A)	0.36 (18:50)	0.15
*SH3BGRL3*	SH3 domain binding glutamic acid-rich protein-like 3	0.12 (0:8)	0.44
*MAPKAPK3*	Mitogen-activated protein kinase-activated protein kinase3	0.11 (0:9)	0.56
*ILK*	Integrin-linked kinase	**1 (4:4)**	**0.64**
*PLCB2*	Phospholipase C, beta 2	0.16 (0:6)	0.40
*PTPRC*	Protein tyrosine phosphatase, receptor type, C	0.5 (3:6)	0.41
*SH3BP2*	SH3-domain binding protein 2	0.44 (4:9)	0.66

**Transcription-related activity**			

*IRF1*	Interferon regulatory factor 1	0.28 (2:7)	0.50
*IRF7*	interferon regulatory factor 7	8 (8:1)	1.37
*STAT1*	Signal transducer and activator of transcription 1	0.22 (4:18)	0.63
*DDX3X*	DEAD (Asp-Glu-Ala-Asp) box polypeptide 3	0.81 (9:11)	0.66
*CEBPB*	CCAAT/enhancer binding protein (C/EBP), beta	**12 (12:1)**	**0.81**
*EGR1*	Early growth response 1	**4.5 (18:4)**	**0.61**

**Lysosomal-associated protein**			

*CTSS*	Cathepsin S	0.15 (3:20)	0.58
*LAPTM5*	Lysosomal-associated protein	**1 (17:17)**	**0.52**
*IFI30*	Interferon, gamma-inducible protein 30	0.58 (67:115)	0.53

The expression of 42 gene transcripts, identified by SAGE analysis, were tested using quatitative PCR. Results show the occurrences (between parentheses) and the ratio of "MDM+Lm"/MDM tags. The last column shows the 2^-ΔΔCt ^values, obtained by quantitative PCR.

On the whole, data generated by SAGE or Q-PCR showed a good concordance between the trends (up- or down-regulation) of expression ratios for 83% of the genes tested, although the response measured by the two techniques might differ in magnitude. The best correlations between SAGE and Q-PCR data were observed for the genes that were abundantly expressed.

### *L. major *infection induces a discreet but selective change in human MΦ transcripts

**Figure 4:**
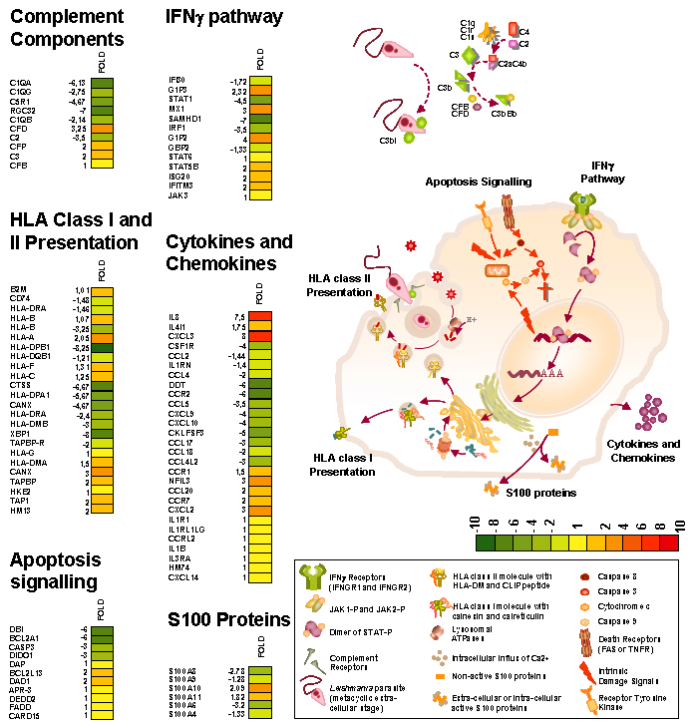
**Examples of gene transcripts categorized into functional classes involved in defense MΦ programs**. This figure shows some gene transcripts related to defense pathways in human macrophages extracted from a list of down- or up-modulated transcripts after exposure to *Leishmania *infection and clustered into functional families (data not shown). Data are reported according to their scale of fold expression values ranging from – 10 (green color) to +10 (red color). For extended names of genes abbreviated see Additional file 5.

Following tag annotation, we used the STRIPE software [28] to screen for any spatial clustering across the human genome (Additional file 4: Spatial Clustering across the Human genome of tags extracted from MDM and "MDM+Lm" libraries). Statistical analysis of transcripts did not show any specific up- or down-modulated gene clustering across the human chromosomes. Further analysis showed that the response of MDM to *Leishmania *infection is characterized by the expression of genes encoding for proteins involved in several biological processes (Figure 4 and Additional file 5: Extended names of abbreviated genes).

#### Complement activation

We first focused our analysis on genes involved in innate immunity such as complement components. *In vivo *opsonization of *Leishmania *promastigotes by C3b and C3bi permits the interaction with the MΦ complement receptors 1 (CR1) and 3 (CR3), respectively. In addition, it is known that C1qA and C1qB molecules are highly up-regulated by activation. Our results showed a drastic inhibition of gene transcription of these latter two proteins upon MDM infection. Several other transcripts, such as *C5R1*, *C2*, *C1qG *or *RGC32*, were also down-regulated after *L. major *infection.

#### S100 proteins

Recently, a novel group of calcium-binding molecules, namely the phagocytic S100 proteins, was described as pro-inflammatory factors. These endogenous damage-associated molecular pattern (DAMP) molecules, also called alarmins, play an important role in innate immunity. Our results showed that *S100A6*, *S100A8 *and *S100A9 *transcripts were repressed upon *L. major *infection. The S100A8/A9 complex has been shown to play an important role in phagocyte NADPH oxidase activation, which contributes to intracellular parasite killing. Their inhibition could be way for *L. major *to avoid reactive oxygen intermediate (ROI) killing. Two other S100 family members were up-regulated (i.e., *S100A10 *and *S100A11*). These two proteins are described as interacting with the N-termini of annexins A1 and A2, forming a sophisticated Ca2+ sensing system. The annexin A2, which acts as a receptor of plasmin, a potent pro-inflammatory activator of human monocytes, was down-regulated twice. The identification of several transcripts of this family modulated by *Leishmania *suggests a novel mechanism of inflammation and tissue damage in infected MΦs.

#### MHC class I and class II molecules

We then investigated major histocompatibility complex (MHC) genes after *L. major *infection. Class II antigen-processing genes, including some cathepsins (i.e., *cathepsin S *or *cathepsin C*) and genes involved in class II presentation such as *CD74*, *Human Leukocyte Antigen *(*HLA*)-*DP*, *HLA-DQ*, *HLA-DR *and *HLA-DM*, were repressed in MDM cells relatively to samples from uninfected cells.

In contrast to the class II pathway, genes involved in antigen processing and presentation via the MHC class I pathway (i.e., *β2-microglobulin*, *tapasin*, *HLA-C*, *HLA-F *and *HLA-G*) were not altered by parasite infection at 24 h, except for *HLA-B *and *calnexin *genes, which were down-regulated, and *HLA-A*, which was up-regulated.

#### Interferon (IFN)γ pathway

Inhibition of the IFNγ pathway appears to be a mechanism that is widely used by different pathogens to subvert the host responses [29]. IFNγ, a potent inducer of MHC class II expression in MΦs and hence of antigen presentation, once bound to its receptor, leads to STAT1 phosphorylation and translocation to the nucleus and to IFN regulatory factor (IRF) activation, which play a key role in the induction of a large set of MΦ effector molecules involved in host defense and inflammation. Our results showed a significant decrease in *IFNGR2*, *STAT1 *and *IRF1 *transcripts in parasite infected MDM.

#### Apoptosis

Programmed cell death plays a pivotal role in normal tissue development and in pathological conditions [30]. Interestingly, *Leishmania *inhibits host cell apoptosis pathways in order to favor its own multiplication [31]. We annotated several tags as apoptotic and anti-apoptotic family members. Transcripts of *caspase 3 *(*CASP3*), *Acyl coenzyme A-binding protein *(*DBI*), *death inducer-obliterator-1 *(*DIDO1*) and *Bcl2-related protein A1 *(*BCL2A1*), are pro-apoptotic proteins or induced by apoptosis, and were down-modulated upon infection. In addition, an anti-apoptotic gene transcript called *defender against cell death-1 *(*DAD1*) was slightly induced.

#### Cytokines and chemokines

We finally focused on cytokine and chemokine transcripts. Several were up-regulated upon infection e.g., *IL-8*, *CXCL2*, *CXCL3 *or *NFIL3*. On the other hand, we noted that the mRNA expression levels of different chemokines and their ligands, i.e., *CCR2*, *CCL5*, *CCL17*, *CXCL9*, *CXCL10*, *CCL4L2 *or *CKLFSF3*, were drastically inhibited upon infection. As expected, the transcripts of the pivotal cytokine IL10 were strongly up-regulated (from 101 to 233 occurrences), even though the assigned tag did not correspond to the tag directly following the poly-A signal. However, we were not able to unambiguously assign tags corresponding to other cytokines, classically reported to be altered after *Leishmania *infection (i.e., *IL12*, *IL18 *or *TNFα*).

### Transcriptome analysis of extra- and intracellular specific stages of *L. major *parasites

Our analysis also included the study of *Leishmania *transcriptome alterations, once parasites were exposed to the phagolysosomal intracellular environment and transform into amastigotes. We focused on (i) the most highly expressed transcripts at the metacyclic stage and (ii) differentially expressed tags between the intracellular and extracellular stages of *L. major *parasite.

### Annotation of *L. major *tags from the metacyclic parasite SAGE library

A total of 33,906 tags corresponding to 9,530 unique tags were generated from the metacyclic stage of the *L. major *library (Table 1). The 106 most abundant ones represented 1.1% of the total number of unique tags (106/9,530) but totaled up to 40% of the entire collection of parasite tags (13,636/33,906).

Tag-to-gene mapping was done for these most abundant (Table 3) and total tags of the *L. major *GLC94 library transcripts using BLAST against the Friedlin *L. major *genome. This snapshot of the major parasitic transcripts showed that 35 out of 106 tags (33%) mapped unambiguously to their genes with 100% sequence identity (downstream stop codon). Twenty-seven tags mapped to a unique gene and 8 mapped to two or more genes belonging to the same family. Among these assigned tags, 32 tags were located in the 3' region, downstream the stop codon and three matched inside the CDS. Finally, from the tags present at least twice (3,163 tags), we were able to assign 1,068 tags to their genes (Additional file 6: "Tag to gene assignation" of all parasitic tags present at least twice and extracted from Lm library).

**Table 3:** The most abundant and annotated transcripts expressed in metacyclic *L. major *grown in culture.

**Tag**	**Absolute Occurrences in Lm library**	**Access number**	**Protein name**
CATGAGCGACCACC	**329**	LmjF31.2030;LmjF31.1900	ubiquitin-fusion protein
CATGATGGGGCGCT	**266**	LmjF35.1890	60S ribosomal protein L5, putative
CATGTCATTTCTCG	**206**	LmjF22.0030	60S ribosomal protein L11 (L5, L16)
CATGTATGTGCGCC	**163**	LmjF36.0600	ubiquitin/ribosomal protein S27a
CATGAGGCACTGTG	**149**	LmjF16.0600	histone h3, putative
CATGCGCGGCAGAC	**135**	LmjF13.0280 to 13.0390	alpha tubulin
CATGGCAACTGTCG	**127**	LmjF36.3740	60S ribosomal protein L34, putative
CATGTTATTTGGCC	**123**	LmjF36.2860;LmjF36.2870	40S ribosomal protein S24e
CATGTCAAATTTGT	**115**	LmjF33.0792 to 33.0819	beta-tubulin
CATGTAATTGACTC	**113**	LmjF29.2370	60S ribosomal protein L39, putative
CATGCGTCCACCGC	**111**	LmjF24.2080	40S ribosomal protein S8, putative
CATGGTTCGCGTGT	**110**	LmjF35.0600	60S ribosomal protein L18a, putative
CATGCCGCATCACT	**108**	LmjF36.0990	40S ribosomal protein S10, putative
CATGGTGTGCAGGT	**99**	LmjF28.2560	40S ribosomal protein S17, putative
CATGTGACCCGTAT	**98**	LmjF31.0900	Hypothetical protein, conserved
CATGGCGTGCATTG	**92**	LmjF11.1110;LmjF11.1130	60S ribosomal protein L28, putative
CATGTGTGCGGATC	**84**	LmjF25.1190	ribosomal protein S25
CATGCCACTTGTTT	**83**	LmjF35.2190	60S ribosomal protein L12, putative
CATGAAGCTTCTGT	**81**	LmjF35.3290	60S ribosomal subunit protein L31
CATGGACGGTAGGC	**69**	LmjF29.1090	ribosomal protein L1a, putative
CATGGACCCGGACG	**67**	LmjF15.0950	40S ribosomal protein S3, putative
CATGCGCGGCCAGA	**64**	LmjF32.2690	ribosomal protein L27, putative
CATGCAAGCGAGGA	**62**	LmjF21.1070	40S ribosomal protein S23, putative
CATGCCCGCAGTAC	**59**	LmjF27.1190	histone H1, putative
CATGAATGCATCTT	**59**	LmjF34.2900	ribosomal protein l3, putative
CATGTGTACAGCCC	**57**	LmjF19.0820 to 19.0900	microtubule associated protein-like protein
CATGTGCAAGACTC	**51**	LmjF34.0440	ribosomal protein S25
CATGGCTTGCTGTG	**50**	LmjF12.0340	Hypothetical protein, unknown function
CATGCGACGAAAGA	**50**	LmjF29.1730	histone H2A, putative
CATGTATGCGTTTT	**49**	LmjF36.3620	Hypothetical protein, conserved
CATGGGCGGTCTCT	**49**	LmjF27.1390;LmjF27.1380	60S acidic ribosomal subunit protein
CATGAGTGGCGAGG	**42**	LmjF21.1550	40S ribosomal protein S11, putative
CATGCGCAGCATCC	**41**	LmjF27.1380	60S acidic ribosomal subunit protein
CATGACAAATAGTC	**41**	LmjF13.0450	Hypothetical protein, conserved
CATGATGCTGCCGC	**41**	LmjF06.0410;LmjF06.0415	60S ribosomal protein L19, putative

This table shows annotated tags from the 106 most abundant tags, their occurrences in the Lm library, their corresponding access numbers and protein names in GeneDB. A table with all annotated *Leishmania *tags are available as Additional file 6.

Through this annotation, several transcripts were found encoding for ribosomal proteins including 40S, 60S, L1a, L27, I3, S25 and S27a ribosomal proteins. This analysis also revealed the abundant expression of mRNA encoding for histones H1, H2A and H3, for ubiquitin related proteins, tubulin and microtubule associated protein among others (Table 3).

### Preferential expression of transcripts in *L. major *parasites at the intracellular stage

When comparing libraries, we found 3,666 tags (2,960 with more than one copy) of them co-expressed in the "MDM+Lm" and Lm, but absent in other human libraries (Figure 1B and 1D). Statistical analysis and fold increase levels revealed that 697 of these tags were differentially expressed between metacyclic promastigotes (Lm library) and intracellular parasites ("MDM+Lm" library), with 420 tags preferentially expressed by intramacrophagic parasites (*p *< 0.05 for 193 and *p *≥ 0.05 for 227 of them but with a fold increase greater then 3.5-fold).

Tag-to-gene mapping of these 420 tags showed that 113 (27%) of them were unambiguously mapped to their genes. Among them, only 105 tags mapped to a unique gene and eight mapped to genes within the same family.

This analysis also revealed differential expression of mRNA encoding for different proteins including an amastin-like protein, histones H1 and H2B, tubulin tyrosine ligase, reiske iron-sulfur protein precursor and several ribosomal proteins (Table 4). Interestingly, 71 of the assigned tags corresponded to hypothetical proteins with conserved domains and/or unknown functions.

**Table 4:** *L. major *annotated transcripts preferentially expressed in human infected MDMs.

**Tag**	**Occurrence**	IP 2^-ΔΔCT^	AP 2^-ΔΔCT^	**Accession number**	**Protein name**
CATGACAATCTTGT	1:4	ND	ND	LmjF01.0490	Long chain fatty acid CoA ligase, putative
CATGGCAGTTATCT	18:36	ND	ND	LmjF04.0750	60S ribosomal protein L10, putative
CATGGGCGTCGCGC	32:48	ND	ND	LmjF08.0670to08.0760	amastin-like protein
CATGCCGGTGGGCC	4:14	ND	ND	LmjF15.1040 to 15.1160	tryparedoxin peroxidase
CATGCCAGCGGGAT	5:16	3.2 ± 0.11	9.4 ± 0.46	LmjF17.1220	Histone H2B
CATGGTAATCCAAA	1:6	ND	ND	LmjF20.0870	ATP-dependent RNA helicase, putative
CATGGTGTAGAGGA	2:6	ND	ND	LmjF22.0110	GMP synthase, & glutamine transferase
CATGAGCGCCTGAA	1:5	ND	ND	LmjF24.1090	Predicted multipass transmembrane
CATGACGGTACCGT	1:5	ND	ND	LmjF26.0210	Silent information regulator 2
CATGCGCGAACTAG	1:4	ND	ND	LmjF26.1700	Fatty acid desaturase, putative
CATGCTAACGTTCT	1:21	1.3 ± 0.09	0.8 ± 0.16	LmjF26.1710	Cytochrome c oxidase subunit V, putative
CATGCGACTTCGCT	1:4	ND	ND	LmjF26.2220	Ribosomal protein l38, putative
CATGGCTGTATCTC	2:21	ND	ND	LmjF26.2220	Ribosomal protein l38, putative
CATGGCTTCCCTCG	27:44	ND	ND	LmjF26.2330	60S ribosomal protein L35, putative
CATGGCGCAGTCCC	1:4	ND	ND	LmjF26.2400	Peroxisomal membrane protein 4, putative
CATGATGGCGCACC	1:5	ND	ND	LmjF27.1190to27.1240	histone H1, putative
CATGACGTGCTTGC	1:6	ND	ND	LmjF27.2320	Protein phosphatase-like protein
CATGCGTAGACGAT	25:64	1.6 ± 0.25	0.5 ± 0.05	LmjF28.2205	Ribosomal protein S29, putative
CATGCTTGCAGCAG	1:4	ND	ND	LmjF29.0630	BET1-like protein, putative
CATGACCGCTGCTA	8:22	ND	ND	LmjF30.0680	40S ribosomal protein S30, putative
CATGTTGTTGTATA	1:6	ND	ND	LmjF31.1960	Tryparedoxin-like protein
CATGGATGGTGTCT	4:13	1.7 ± 0.2	5 ± 0.45	LmjF31.2250	3,2-trans-enoyl-CoA isomerase
CATGCGCGAATAGG	2:6	ND	ND	LmjF31.2850	Ribosomal protein l7/l12-like protein
CATGGCGTAGAGAA	1:5	ND	ND	LmjF31.3130	Methylcrotonoyl-coa carboxylase protein
CATGCGGAGTCAGG	1:7	3 ± 0.22	1.6 ± 0.09	LmjF33.0260	RNA binding protein rggm, putative
CATGGCCGGATAGA	1:4	ND	ND	LmjF33.2300	udp-glc 4'-epimerase, putative
CATGTCGGCGGTGA	1:8	2.3 ± 0.21	2.44 ± 0.16	LmjF34.3430	Cleavage & polyadenylation factor
CATGGCCGCACCGG	2:9	ND	ND	LmjF34.365 & 16.0460	60S ribosomal protein L21, putative
CATGCACACAGCGA	1:4	ND	ND	LmjF35.1470	choline/ethanolamine kinase, putative
CATGTGTGCTCTTA	2:8	0.9 ± 0.08	1.5 ± 0.08	LmjF35.1540	Reiske iron-sulfur protein precursor
CATGGGTAAGGATC	3:10	ND	ND	LmjF35.3790to35.3800	60S ribosomal protein L23, putative
CATGTCGATACCCG	1:8	1.9 ± 0.08	2 ± 0.13	LmjF35.4930	Tubulin tyrosine ligase, putative
CATGTCCAGTGGGA	2:8	ND	ND	LmjF35.5100	60S ribosomal protein L37
CATGTGTACGAGTC	1:11	ND	ND	LmjF36.0010&25.2460	galactosyltransferase 1, 2, 3, 4 & 6
CATGTGATCTTCCG	2:9	2.4 ± 0.06	2,1 ± 0.13	LmjF36.0070	Stress-inducible protein STI1 homolog
CATGCTGCGGTGTA	1:4	ND	ND	LmjF36.1050	RNA editing complex protein MP61
CATGCGCAAGAAGA	1:4	ND	ND	LmjF36.1070to36.1100	ribosomal protein L24, putative
CATGGGGACGCGTT	7:20	1.3 ± 0.07	0.5 ± 0.03	LmjF36.1250	40S ribosomal protein S9, putative
CATGCCCACCACGC	17:28	ND	ND	LmjF36.3390	Ribosomal protein L29, putative
CATGCTTGTGTGAC	12:23	1.4 ± 0.19	0.9 ± 0.05	LmjF36.3760	60S ribosomal protein L10a, putative
CATGGTGGTGTATG	1:4	ND	ND	LmjF36.3810	Aminomethyltransferase
CATGCTGCGGCGGT	1:6	2.6 ± 0.71	2.3 ± 0.17	LmjF36.5880	Small GTPase, putative

This table shows annotated tags from the 420 differentially expressed tags at the intracellular *L. major *stage, their occurrences in the Lm and "MDM+Lm" SAGE libraries and their corresponding access numbers and protein names in GeneDB. Underlined accession numbers correspond to genes tested by quantitative RT-PCR; results are presented as mean 2^-ΔΔCt ^values ± SD obtained with intracellular (IP) or with amastigote-like axenic (AP) parasites. ND: non-determined.

### Stage-specific preferential expression of parasite transcripts is confirmed by quantitative PCR experiments

We used quantitative real-time PCR to validate the accuracy of the SAGE data generated. Q-PCR was also performed on cDNA obtained from amastigote-like axenic parasites of *L. major*.

By comparing "MDM+Lm"/MDM tags ratios, Q-PCR showed the same trend towards the up-regulated expression of all selected transcripts, but one, in intracellular parasites compared to *L. major *promastigote metacyclic parasites (Table 4). Unexpectedly, 33% of the tested transcripts that were up-regulated in intracellular amastigotes, using SAGE and Q-PCR technologies, were down-regulated in the amastigote-like parasites obtained by culture in axenic conditions. This result suggests that the transcriptome profile of *L. major *amastigote-like axenic parasites may not reproduce the profile expressed by the naturally induced intracellular amastigote stage and that the biological results obtained with the former parasite should be cautiously extrapolated to the latter parasite form.

## Discussion

Genome-wide expression profiling offers new perspectives for studying host-pathogen interactions to decipher, at the transcriptional level, how host cells react to infection and how pathogens adapt to their host's microenvironment. In the present study, we took advantage of SAGE to analyze the transcriptomes of both the infected MΦ and the intracellular parasite *Leishmania *using a one-step approach. Our working hypothesis was that, having extracted the bulk of mRNA molecules from a co-culture of parasites and infected MΦs, it would be possible to separate, in the resulting SAGE library, the respective contributions of each organism to the mixed collection of tags. The proportion of ambiguous gene signatures was found to be lower than 1.5%, confirming the validity of this approach. Such unambiguous tag species identification would be more difficult to reach using alternative high-throughput transcriptomic methods, such as microarrays, due to the difficulties in assessing the extent of cross-hybridization between the human and the parasite transcripts.

Separating the contribution of both organisms in an infected MΦsAGE library raised no technical problems and could be performed on a desktop computer using the functions of a commercial database management system (MS-Access). To distinguish tags according to their origin, we considered that merging all publicly available leukocyte libraries would generate a set of tags that are representative of human transcripts. Despite this extended coverage, it is clear that the deconvolution of both transcriptomes could not be complete, since unmatched tags that could not be ascribed to either of the two species (*H. sapiens or L. major*) may either correspond to very specific human transcripts expressed only in *Leishmania*-infected MΦs and never generated elsewhere or may reveal stage-specific parasite transcripts strictly specific of the intracellular stage. This problem was pointed out in a recent study [32], suggesting that the human genome might actually contain twice as many transcribed regions as currently annotated. Moreover, the ENCODE project consortium highlighted the number and complexity of the RNA transcripts generated comparatively to the small number of protein-coding genes (≈ 21,000) currently annotated on the human genome [33,34].

SAGE was used as a quantitative approach, to evaluate the expression levels of mRNAs and to calculate the respective amount of material from human or parasite origin. With the reasonable assumption that mRNAs originated only from living cells, our data demonstrated the importance (49%) of the parasitic load in infected cells.

In spite of this heavy parasitic burden, a salient feature emerged from multivariate statistics: that parasite infection has, at the global level, an apparent marginal impact (only 2.4% of the transcripts were found modulated) on the expression profile of infected MΦs. Thus, the mRNA profile of infected MΦs contrasted with that of monocytes exposed to LPS because it revealed many fewer alterations in gene expression.

However, although *Leishmania *parasites do not seem to induce dramatic changes in the transcriptional remodeling program of MΦs, a closer analysis detected physiologically significant alterations in gene transcription. Despite their discreetness, these alterations could harmfully weaken macrophages' microbicidal defense task and homing properties. Indeed, our analysis showed that several MΦ antiparasitic pathways were altered at the level of mRNA expression upon infection by *L. major *parasites. In particular, we were able to show that several members of the S100A family, among others, are up- or down-regulated by infection. This is in contrast to a previous study using microarray technology that reported almost stable signals between non-infected MΦs as compared to *L. major*-infected MΦs for this gene family [10]. Other differences in the expression levels of several chemokine family members were observed between the two studies, except for *CXCL3 *and *IL8 *transcripts, which were strongly up-regulated.

Whether the discrepancies between the two approaches reflect differences in the experimental protocols used by the two studies (e.g., cell-parasite incubation time, parasite strains or human genetic variability) or are attributable to differences in the sensitivity of the two techniques to accurately quantitate the mRNA of expressed genes is unclear. It is noteworthy that our results concerning the IFNγ pathway, are in agreement with those obtained recently by Dogra et al. in THP1-infected cells [11]. Indeed, transcripts of *STAT-1*, a key actor of this pathway, were drastically down-regulated at 24 h after infection, though there is no external activation by IFNγ. In addition, we found that several IFNγ-inducible chemokines (CXCL9 and CXCL10) were down-modulated. Since key proteins belonging to this pathway are also inhibited upon *L. major *infection (K Ben-Aissa, Personal communication), such effects render the MΦ refractory to any potential activation by IFNγ and obviously favor parasite survival. Other genes, among those involved in antigen presentation and implicated in the stabilization and the recycling of classical MHC class II and in the binding and the capture of antigens were also down-modulated by *L. major*, as reported by Chaussabel et al. and Dogra et al. [10,11].

Our results also show that several genes encoding pro-inflammatory mediators were up-regulated, while other family members were down-modulated. This indicates that *Leishmania *have a remarkable capacity to specifically inhibits the transcription of several molecules associated with pro-inflammatory responses. It is notable that this peculiarity of *L. major *infection does not completely fit – in contrast to other pathogens (i.e., *Mycobacterium tuberculosis*, *Listeria monocytogenes*, *Escherichia coli*, *Bordetella pertussis*, *Candida albicans*, *etc*.) – with the so called "common host-transcriptional response" [35], stressing the particularity of this parasite. This is probably a survival mechanism whereby the parasites can inhibit a harmful inflammatory reaction in order to slip silently into the MΦ and successfully establish inside the host.

In addition to the analysis of the MΦ transcriptome, in the last 5 years, several studies have focused on the parasitic transcriptome taking advantage of the availability of *L. major *genome sequence [36]. Although this genome (35 Mb distributed across 36 chromosomes of varying lengths i.e., 0,3 to 2,8 Mb, and coding for roughly 8,370 manually annotated protein-coding genes), was declared to be finished in 2005 [36], only 2,191 *L. major *ESTs originating from cDNA libraries of various sources, such as promastigote or amastigote full length cDNA libraries, are reported on NCBI.

Hence, our tag-to-gene mapping for parasite transcripts was rather encouraging, compared to the number of sequenced tags. Indeed, we were able to list up to 900 tags expressed in at least two copies in the metacyclic promastigote stage but totally absent from the intracellular amastigote stage, generating useful data for better data mining. In addition, among the tags common to *L. major *promastigote and MΦ-infected libraries, 19% (697/3,666) were differentially expressed. This led us to estimate (without taking into account the tags that were specifically intracellular and present only in the infected MΦ library) the transcripts differentially expressed, between the two parasitic stages, to roughly 1,600 tags, representing approximately 20% of transcripts if one considers the 8,370 annotated *Leishmania *genes registered in the databases.

This figure is several-fold higher than those reported from a variety of *Leishmania *species (i.e., *L. major *[13-15,18], *L. donovani *[20], *L. infantum *[17] and *L. mexicana *[16]), which clearly show limited differences using microarrays (ranging from 0.2 to 5% of total genes) in stage-specific gene expression between the promastigote and amastigote life stages. These studies also show that the vast majority of genes are constitutively expressed [18,20,37]. One should note that these studies analyzed the amastigote transcripts, either using amastigote parasites derived from BALB/c lesions or axenic amastigotes obtained *in vitro*, whereas our study used the amastigotes derived from human MΦs.

However, while analyzing the functional significance of gene expression in *Leishmania*, we should consider that it is mainly regulated at the post-transcriptional level. As highlighted by Cohen-Freue et al. [37], the alteration in mRNA levels of regulated genes in *Leishmania *does not necessarily correlate with subsequent protein abundance. The functional significance is better manifested at the protein level, which is regulated by mechanisms such as stage-specific translational control, RNA stability, processing events and post-translational modifications. Nonetheless and despite these limitations, transcriptomic approaches for *Leishmania *could mainly help to better annotate its genome and to study the stability and translational regulation of its transcripts.

## Conclusion

To our knowledge, we provide here for the first time a large-scale gene expression profile of both the infected human MΦ and the infective form of *L. major *using SAGE. This set of expressed genes deserves future rounds of data mining and experimental work, since it contains latent information about proteins susceptible to behaving as antigens and being evaluated as candidates in a vaccine approach. These data also provide the basis for studies in progress that aim to compare, at the molecular level, various strains of *Leishmania *known to differ by their behavior at the physiopathological level. Thus, comparing viscerotropic strains, e.g., *L. infantum *or *L. donovani*, to strictly dermotropic strains e.g., *L. major*, may reveal differences at the level of parasite-MΦ interactions that could indicate cellular targets of parasite virulence factors as well as decipher mechanisms of specific tissular tropism.

## Methods

### Parasite culture and preparation

*L. major *isolate obtained from the field (MHOM/TN/95/GLC94) was used [38]. Parasites were cultured at 26°C without CO_2 _in endotoxin-free RPMI 1640 medium supplemented with 10% heat-inactivated fetal calf serum (HyClone Laboratories, Logan, UT, USA), 100 U/ml penicillin, 100 μg/ml streptomycin and 2 mM L-glutamine. Infective-stage metacyclic promastigotes were isolated from stationary culture (5–6 days old) by negative selection using peanut agglutinin (Sigma, Saint-Quentin Fallavier, France). Parasites were then harvested for RNA preparation or used to infect cells (5:1 parasite-to-cell ratio). Axenic amastigotes of *L. major *were obtained by shifting the incubation conditions of a saturated culture of *L. major *promastigotes from 26 to 37°C and pH 5.5 in a modified RPMI 1640 medium as described previously [39].

### *In vitro *generation of human MΦs

Donors were selected as negative for any recent infection and with no history of Leishmaniasis. Their peripheral blood mononuclear cells (PBMC) did not proliferate *in vitro *to Soluble *Leishmania *Antigens and they were not taking medication at the time of the study. Informed consent was obtained from all donors. The experimental protocol was approved by the institutional ethics committee of the Institute Pasteur of Tunis. Human PBMCs were isolated from leukopack peripheral blood mononuclear cells of four healthy volunteers using Ficoll-Paque (Pharmacia, Uppsala, Sweden) density gradient centrifugation. Cells were washed and resuspended at 10^6 ^cells/ml in RPMI 1640 medium supplemented with 2 mM L-glutamine, 100 U/ml penicillin, 100 μg/ml streptomycin and 10% autologous heat-inactivated serum. Monocytes were purified by fibronectin-mediated adhesion [40] using gelatin (Sigma) and autologous heat-inactivated serum substratum.

Monocyte cell purity was assessed by flow cytometry (FACSVantage; Becton Dickinson, Sunnyvale, CA, USA) using directly conjugated anti-CD3, anti-CD19 and anti-CD14 antibodies (Becton-Dickinson, San Jose, CA, USA) and was routinely greater than 85% of CD14+ cells.

To obtain MΦs, monocytes were cultured for 8 days at 37°C, 5% CO_2 _in endotoxin-free RPMI 1640 medium supplemented with 10% heat-inactivated normal human AB serum and 10% heat-inactivated fetal calf serum (HyClone Laboratories), 100 U/ml penicillin, 100 μg/ml streptomycin, 2 mM L-glutamine at 2× 10^6 ^cells/ml, in six-well tissue-culture plates.

### Human MΦs infection

The MDMs obtained were exposed to metacyclic parasites of *L. major *(MHOM/TN/95/GLC94 strain parasites to cells (ratio 5:1) for 24 h and then harvested for RNA preparation. To determine infection levels, an aliquot was taken from each culture, spun onto glass microscope slide, and stained with Giemsa-May Grünwald. The percentage of infected cells was counted by microscopy, in triplicate of one hundred cells for each slide.

### RNA isolation

Cells or parasites were collected at the indicated time points by centrifugation, homogenized by Trizol reagent (Gibco BRL) and frozen at -70°C until RNA extraction. The RNA from each of the four donors was extracted independently then pooled, and used for library construction.

RNA was purified from contaminating genomic DNA using the standard protocol. Briefly, contaminating DNA was removed from total macrophage or parasite RNA using *DNase I *(Invitrogen, Carlsbad, CA, USA). The RNA samples were then ethanol-precipitated, washed once in 70% ethanol, and redissolved in water. RNA was quantified using a spectrophotometer. Examination of purified total RNA by gel electrophoresis revealed prominent 5S, 18S and 28S ribosomal bands for human samples and 18S and 24Sα and 24Sβ ribosomal bands for parasitic samples, indicating that the RNA was not degraded.

### SAGE library construction

Libraries were constructed using the I-SAGE Kit (Invitrogen) according to the protocol developed by Velculescu et al. [41]. Briefly, a pool of mRNA samples was converted into cDNA using biotinylated oligo(dt) primer linked to magnetic beads. The cDNA were cleaved using the *NlaIII *anchoring enzyme. Digested DNAs were split in two and each ligated with one of two adapters containing a restriction site of *BsmFI *tagging enzyme. The two pools of the tags obtained were ligated to one another and served as templates for PCR amplification. The PCR product (containing two tags (ditag) linked tail to tail) was then cleaved with the *NlaIII *anchoring enzyme, thus releasing 14 bp-long ditags that were then concatenated by ligation, cloned and sequenced.

### Computer-based analysis of the SAGE libraries

The raw sequences obtained from concatemer clones were analyzed using PHRED [42] and trimmed for quality to eliminate erroneous tags as much as possible. Contaminating vector sequences or SAGE tags derived from linkers were then discarded using CROSS-MATCH software [43]. Experimental tag sequences were extracted using DIGITAG [26]. This software is written in PERL and implemented on a UNIX operating workstation for automatic tag detection and counting. DIGITAG analyzes all concatemer sequences to discard ditags that are duplicated or different from 20 bp between the two CATG. Then, for each concatemer sequence, DIGITAG generates the reverse complement, adds it to the initial sequence, and extracts all CATGs plus the 10 following bases to obtain the tag sequences and determine their copy number in each library. *P*-value calculations and identification of genes differentially expressed were performed according to the procedure described by Piquemal et al. [26]. Tag levels were compared between the two MDM libraries generated in the absence or presence of parasites, or between the MDM-infected library and metacyclic parasite library. Differentially expressed tags were selected for further analysis. Expression data were also analyzed using various modules of the TIGR MultiExperiment Viewer Package (MeV 4.0, 2006).

### Human leukocyte SAGE library collections

Following sequence analysis of SAGE libraries, data were assembled in a unique matrix. We also collected up to 357,888 experimental tags from nine publicly available human leukocyte SAGE libraries (retrieved from [44-46]) and from a second in-house non-stimulated MΦ generated library (raised in similar conditions but from a different pool of donors and noted MDM-M; Ottones et al., unpublished data). These SAGE libraries were generated from freshly isolated monocytes [47], M-CSF-differentiated [47], GM-CSF-differentiated [47] and LPS-activated [48] cells, immature [49] and mature [50] monocyte-derived dendritic cells and unfractionated populations of leukocytes [51], noted Mono, M-CSF, GM-CSF, LPS, IDC, MADC, leuk, WBC-N and WBC-Bc respectively. Data were assembled to build a matrix giving the expression levels of 26,176 unique tags.

### Human tag-to-gene mapping

Regular SAGE tags were identified as previously described [26]. Briefly, we constructed a reference database to compile tags predicted from collections of expressed sequences, including well-annotated sequences [52], reference sequences of UniGene clusters [53], SAGEmap tags [44] and the GenBank collection of human Alu sequences. This Preditag^® ^software (Skuld-Tech, Montpellier, France) was also modified to register virtual tags matching the reverse complement of the sequences. We used its functions to automatically generate a table of results, by matching experimental tags to virtual ones. Tags matching with 100% sequence identity were then ranked based on the fidelity of the source sequence. The first positions starting from the 3'-most end of the transcript were kept.

### Parasite tag-to-gene mapping

In order to assign gene identity to each parasite tag, the experimental tag list from the purified metacyclic parasite SAGE library were matched against the *L. major *Friedlin genome (version 5.2) downloaded from GeneDB [54].

Since SAGE tags should be sitting in the untranslated regions of a given gene, tags that had a unique match, with 100% sequence identity, and that were found within 1 kb downstream of the stop codon of one gene or related genes within the same family and alternatively in the CDS, were assigned. These related genes were identified by blast, comparing the set of *L. major *proteins and selecting those that were sharing more than 85% identity.

### Validation of SAGE libraries by Q-PCR

The same pooled RNAs used for SAGE libraries were used for real-time reverse transcriptase-polymerase chain reaction (RT-PCR). For human tag validation analyses, predeveloped assay reagent probes, reagents, and Real-time PCR ABI-7900HT equipment were used for validation experiments as recommended by the manufacturer (Applied Biosystems, Fullerton, CA, USA). For parasite tag validation studies, reverse transcription and real-time PCR were performed using SYBR Green I Universal PCR MasterMix (PE Applied Biosystems, Foster City, CA, USA) and primers (Additional file 7: Parasite primer sequences for quantitative RT-PCR) were designed for each sequence, including endogenous controls, using Primer express Software (Version 1.5, PE Applied Biosystems). All PCR reactions were performed using the ABI PRISM 7700 sequence detection system. This technique is based on measuring PCR products in the logarithmic phase of the reaction by determining the CT [55], CT being the threshold cycle at which the fluorescence emission reaches the log phase of product accumulation.

Briefly, after defrosting at room temperature, total RNA was extracted using the Qiagen RNeasy Mini kit as indicated by the manufacturer (Qiagen, Courtaboeuf, France). The quality of the total RNA was determined by capillary electrophoresis analysis using an Agilent 2100 Bioanalyser (Agilent, Palo Alto, CA, USA). cDNA was then synthesized using the High-Capacity cDNA Archive Kit (Applied Biosystems) according to the manufacturer's protocol. Samples were loaded on micro-fluidic plates and data were normalized by referring to the expression of an endogenous control which was highly homogeneous between used samples (Ct = 20.55 for MDM library and 20.46 for "MDM+Lm" library, i.e., human glyceraldehyde-3-phosphate dehydrogenase (GAPDH).

For parasite Q-PCR, each analysis was performed in triplicate and data were normalized by referring to the expression of an endogenous control (rRNA45; accession number: CC144545) described as equally expressed between procyclic, metacyclic, and amastigote stages of *L. major *using DNA microarrays, quantitative PCR and Northern blot experiments [14,56].

Finally, for each human target mRNA, results were expressed as a fold difference in MDM exposed to metacyclic parasites of *L. major *promastigotes vs. non-infected MDM by calculating 2^-ΔΔCT^. For parasite target mRNA, results were expressed as a fold difference in *L. major *metacyclic promastigotes vs. *L. major*-infected MDM.

## Abbreviations

CR: Complement receptors; *L*: *Leishmania*; MΦ: Macrophage; MDM: Monocyte-derived macrophages; MDM+Lm: MDM infected with *L. major*; Q-PCR: Quantitative-Polymerase Chain Reaction; SAGE: Serial Analysis of Gene Expression.

## Authors' contributions

FZG and DL participated in the MDM preparation and infection experiments, to SAGE library construction, data analysis and manuscript writing. They did parasitic and human RT-PCR validation. LG-T contributed to the MDM preparation and infection experiments. FO participated in the construction of the two MDM SAGE libraries, the RT-PCR validation of human transcripts and data analysis. KB-A participated in the MDM preparation and infection experiments and parasitic SAGE library construction. OM worked on parasitic SAGE library construction. LM and DP extracted tags from raw sequence data and did statistical analysis. TC participated in the analysis of human RT-PCR data. ABK and SS designed parasitic annotation and realized parasite tag assignation. JM and KD contributed to data analysis and manuscript writing and supervised this work.

All authors read and approved the final version of the manuscript.

## Supplementary Material

Additional File 1 **Unique SAGE tags as a function of total sequenced tags in the different constructed libraries**. This figure show the number of unique tags present in the MDM (black), "MDM+Lm" (red) or Lm (green) libraries as a function of sequenced tags in these libraries. Panel A represents the total sequenced tags and panel B represents tags present at least twice. Click here for file

Additional File 2 **Scatter plot showing the comparison of the MDM versus "MDM+Lm" SAGE libraries**. This figure shows comparison between the MDM and "MDM+Lm" libraries of scaled tag frequencies (size dots relatively to the occurrences) and their statistical significance (color dots relatively to the *p*-values). Click here for file

Additional File 3 **Hierarchical Clustering raised with the 500 most abundant tags (left panel) or with the next 2418 tags (right panel)**. The clustering was done using various modules of the TIGR MultiExperiment Viewer Package (MeV 4.0, 2006). "MDM+Lm" corresponds to macrophage-specific tags exctracted from the sample infected by *Leishmania*, MDM to the library built with the same MDM preparation and MDM-M to a second in-house MDM library prepared independently raised in similar conditions from another pool of donors. Mono, LPS, M-CSF, GM-CSF, IDC, MADC, leuk, WBC-N and WBC-Bc are publicly available SAGE libraries (see Methods' section). Click here for file

Additional File 4 **Spatial Clustering across the Human genome of tags extracted from the MDM and "MDM+Lm" libraries**. This figure shows tags plotted as a function of position across the human genome sequence. Each of the 24 human chromosomes is depicted as a thin line in a 5' to 3' orientation. Down-modulated transcripts are shown as green bars, up-modulated transcripts as red bars and unchanged transcripts as black bars. Click here for file

Additional File 5 **Extended names of abbreviated genes**. this file contains the extended names of genes abbreviated in figure 4 presenting examples of gene transcripts categorized into functional classes involved in defense MΦ programs. Click here for file

Additional File 6 **"Tag to gene assignation" of all parasitic tags present at least twice and extracted from Lm library**. This file contains the tag sequences, their occurrences in Lm library, their corresponding access numbers in GeneDB, their orientation across *L. major *genome and the number of genes they belong to (unique or multiple). Click here for file

Additional File 7 **Parasite primer sequences for quantitative RT-PCR**. This file contains the access numbers in GeneDB of the corresponding tag and the 5' to 3' sequences of the forward and reverse primers used in quantitative PCR experiments. Click here for file
