# Supplementary figures and images for: Simultaneous gene expression profiling in human macrophages infected with Leishmania major parasites using SAGE
Source: BMC Genomics. 2008 May 21;9:238. doi: 10.1186/1471-2164-9-238 (PMC2430024; doi:10.1186/1471-2164-9-238)

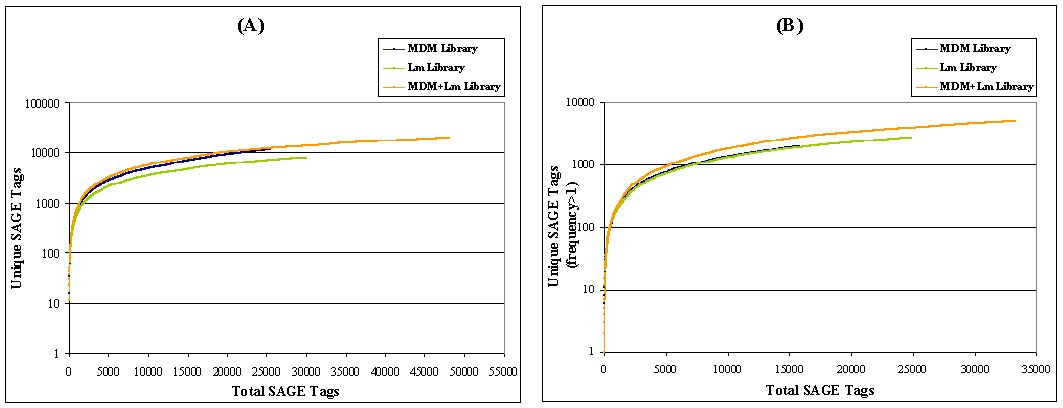

Supplement: Additional File 1 — Unique SAGE tags as a function of total sequenced tags in the different constructed libraries. This figure show the number of unique tags present in the MDM (black), "MDM+Lm" (red) or Lm (green) libraries as a function of sequenced tags in these libraries. Panel A represents the total sequenced tags and panel B represents tags present at least twice. [file 1471-2164-9-238-S1.tiff]

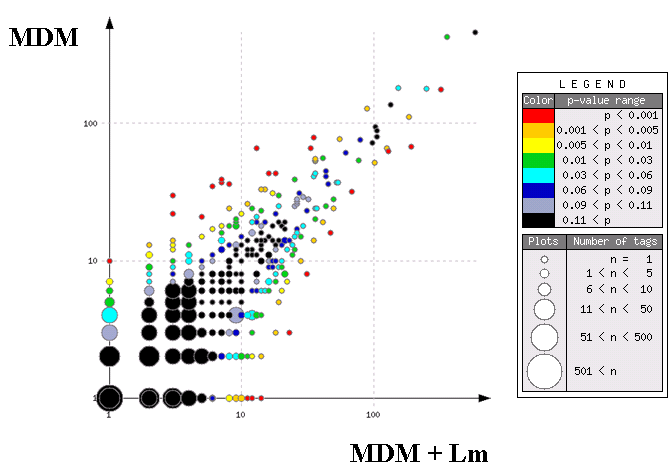

Supplement: Additional File 2 — Scatter plot showing the comparison of the MDM versus "MDM+Lm" SAGE libraries. This figure shows comparison between the MDM and "MDM+Lm" libraries of scaled tag frequencies (size dots relatively to the occurrences) and their statistical significance (color dots relatively to the p-values). [file 1471-2164-9-238-S2.tiff]

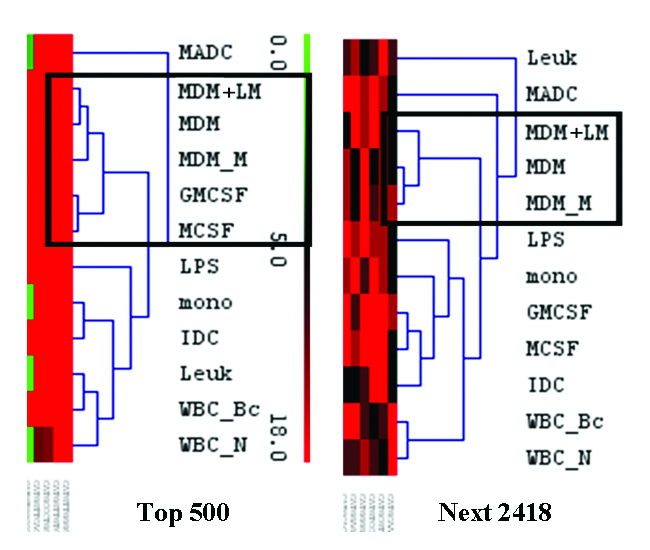

Supplement: Additional File 3 — Hierarchical Clustering raised with the 500 most abundant tags (left panel) or with the next 2418 tags (right panel). The clustering was done using various modules of the TIGR MultiExperiment Viewer Package (MeV 4.0, 2006). "MDM+Lm" corresponds to macrophage-specific tags exctracted from the sample infected by Leishmania, MDM to the library built with the same MDM preparation and MDM-M to a second in-house MDM library prepared independently raised in similar conditions from another pool of donors. Mono, LPS, M-CSF, GM-CSF, IDC, MADC, leuk, WBC-N and WBC-Bc are publicly available SAGE libraries (see Methods' section). [file 1471-2164-9-238-S3.tiff]

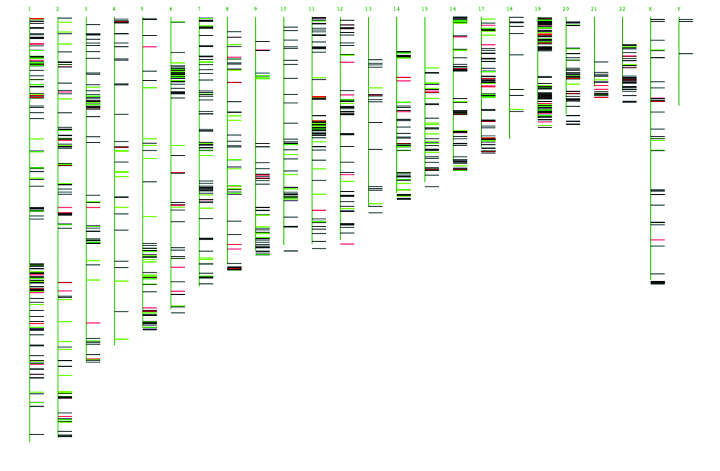

Supplement: Additional File 4 — Spatial Clustering across the Human genome of tags extracted from the MDM and "MDM+Lm" libraries. This figure shows tags plotted as a function of position across the human genome sequence. Each of the 24 human chromosomes is depicted as a thin line in a 5' to 3' orientation. Down-modulated transcripts are shown as green bars, up-modulated transcripts as red bars and unchanged transcripts as black bars. [file 1471-2164-9-238-S4.tiff]
