# Supplementary material for: Simultaneous gene expression profiling in human macrophages infected with Leishmania major parasites using SAGE
Source: BMC Genomics. 2008 May 21;9:238. doi: 10.1186/1471-2164-9-238 (PMC2430024; doi:10.1186/1471-2164-9-238)
Supplement: Additional File 5 — Extended names of abbreviated genes. this file contains the extended names of genes abbreviated in figure 4 presenting examples of gene transcripts categorized into functional classes involved in defense MΦ programs. [file 1471-2164-9-238-S5.doc]

**Additional file 5: Extended names of genes abbreviated in figure 6**

**CYTOKINES AND CHEMOKINES**

GENE_SYMBOL GENE_NAME

IL8 interleukin 8

IL4I1 interleukin 4 induced 1

CXCL3 chemokine (C-X-C motif) ligand 3

CSF1R colony stimulating factor 1 receptor, formerly McDonough feline sarcoma viral (v-fms) oncogene homolog

CCL2 chemokine (C-C motif) ligand 2

IL1RN interleukin 1 receptor antagonist

CCL4 chemokine (C-C motif) ligand 4

DDT D-dopachrome tautomerase

CCR2 chemokine (C-C motif) receptor 2

CCL5 chemokine (C-C motif) ligand 5

CXCL9 chemokine (C-X-C motif) ligand 9

CXCL10 chemokine (C-X-C motif) ligand 10

CKLFSF3 chemokine-like factor super family 3

CCL17 chemokine (C-C motif) ligand 17

CCL18 chemokine (C-C motif) ligand 18 (pulmonary and activation-regulated)

CCL4L2 chemokine (C-C motif) ligand 4-like 2

CCR1 chemokine (C-C motif) receptor 1

NFIL3 nuclear factor, interleukin 3 regulated

CCL20 chemokine (C-C motif) ligand 20

CCR7 chemokine (C-C motif) receptor 7

CXCL2 chemokine (C-X-C motif) ligand 2

IL1R1 interleukin 1 receptor, type I

IL1RL1LG interleukin 1 receptor-like 1 ligand

CCRL2 chemokine (C-C motif) receptor-like 2

IL1B interleukin 1, beta

IL3RA interleukin 3 receptor, alpha (low affinity)

HM74 putative chemokine receptor

CXCL14 chemokine (C-X-C motif) ligand 14

**COMPLEMENT**

C1QA complement component 1, q subcomponent, alpha polypeptide

C1QG complement component 1, q subcomponent, gamma polypeptide

C5R1 complement component 5 receptor 1 (C5a ligand)

RGC32 response gene to complement 32

C1QB complement C1q subcomponent subunit B precursor

CFD complement factor D (adipsin)

C2 complement component 2

CFP complement factor properdin

C3 complement component 3

CFB B-factor, properdin

**HLA CLASS I AND CLASS II PRESENTATION**

B2M beta-2-microglobulin

CD74 CD74 antigen (invariant polypeptide of major histocompatibility complex, class II antigen-associated)

HLA-DRA major histocompatibility complex, class II, DR alpha

HLA-B major histocompatibility complex, class I, B

HLA-B major histocompatibility complex, class I, B

HLA-A major histocompatibility complex, class I, A

HLA-DPB1 major histocompatibility complex, class II, DP beta 1

HLA-DQB1 major histocompatibility complex, class II, DQ beta 1

HLA-F major histocompatibility complex, class I, F

HLA-C major histocompatibility complex, class I, C

CTSS cathepsin S

HLA-DPA1 major histocompatibility complex, class II, DP alpha 1

CANX calnexin

HLA-DRA major histocompatibility complex, class II, DR alpha

HLA-DMB major histocompatibility complex, class II, DM beta

XBP1 X-box binding protein 1

TAPBP-R TAP binding protein related

HLA-G HLA-G histocompatibility antigen, class I, G

HLA-DMA major histocompatibility complex, class II, DM alpha

CANX calnexin

TAPBP TAP binding protein (tapasin)

HKE2 HLA class II region expressed gene KE2

TAP1 transporter 1, ATP-binding cassette, sub-family B (MDR/TAP)

HM13 histocompatibility (minor) 13 (clone)

**IFNγ PATHWAY**

IFI30 interferon, gamma-inducible protein 30

G1P3 interferon, alpha-inducible protein (clone IFI-6-16)

STAT1 signal transducer and activator of transcription 1, 91kDa

MX1 myxovirus (influenza virus) resistance 1, interferon-inducible protein p78 (mouse)

SAMHD1 SAM domain and HD domain-containing protein 1 (Dendritic cell-derived IFNG-induced protein) (DCIP) (Monocyte protein 5) (MOP-5).

IRF1 interferon regulatory factor 1

G1P2 interferon, alpha-inducible protein (clone IFI-15K)

GBP2 guanylate binding protein 2, interferon-inducible

STAT6 signal transducer and activator of transcription 6, interleukin-4 induced

STAT5B signal transducer and activator of transcription 5B

ISG20 interferon stimulated gene 20kDa

IFITM3 interferon induced transmembrane protein 3 (1-8U)

JAK3 Janus kinase 3 (a protein tyrosine kinase, leukocyte)

**APOPTOSIS SIGNALLING**

DBI diazepam binding inhibitor (GABA receptor modulator, acyl-Coenzyme A binding protein)

BCL2A1 BCL2-related protein A1

CASP3 caspase 3, apoptosis-related cysteine protease

DIDO1 Death inducer-obliterator 1

DAP death-associated protein

BCL2L13 BCL2-like 13 (apoptosis facilitator)

DAD1 defender against cell death 1

APR-3 apoptosis related protein APR-3

DEDD2 Death effector domain containing 2

FADD Fas (TNFRSF6)-associated via death domain

CARD15 caspase recruitment domain family, member 15

**S100 PROTEINS**

S100A8 S100 calcium binding protein A8 (calgranulin A)

S100A9 S100 calcium binding protein A9 (calgranulin B)

S100A10 S100 calcium binding protein A10 (annexin II ligand, calpactin I, light polypeptide (p11))

S100A11 S100 calcium binding protein A11

S100A6 S100 calcium binding protein A6 (calcyclin)

S100A4 S100 calcium binding protein A4 (calcium protein, calvasculin, metastasin, murine placental homolog)
