# Supplementary material for: Simultaneous gene expression profiling in human macrophages infected with Leishmania major parasites using SAGE
Source: BMC Genomics. 2008 May 21;9:238. doi: 10.1186/1471-2164-9-238 (PMC2430024; doi:10.1186/1471-2164-9-238)
Supplement: Additional File 7 — Parasite primer sequences for quantitative RT-PCR. This file contains the access numbers in GeneDB of the corresponding tag and the 5' to 3' sequences of the forward and reverse primers used in quantitative PCR experiments. [file 1471-2164-9-238-S7.doc]

**Additional file 7. Parasite primer sequences for quantitative RT-PCR**.

| **Access number** | **Forward primer (5' to 3')** | **Reverse primer (5' to 3')** |
| --- | --- | --- |
| LmjF17.1220 | TCCCCCACTTCTCCCTCTTTC | GCAATGAGAAGCGGGAGGA |
| LmjF26.1710 | GAGCGGCACCCTCTGTCAC | TCTCTCTGGACCGCCTCACTT |
| LmjF28.2205 | GCCAGCATGGGAGGAGATAGA | TCTGCACACCTGCTGCCTT |
| LmjF31.2250 | CTCCTGCCTTCCTCGTGCT | CGGTCGTATCACATCACCCC |
| LmjF33.0260 | GCAGCGAAACCAGTCATGTTG | GCGTGAGACGAGCCTGTGA |
| LmjF34.3430 | TAGGCCTTGGACGGTCCTG | CCTATACATGGCGCAGACGTG |
| LmjF35.1540 | TGCTGGGTTGTCATTGCGA | CGACGACAACACCCAAGATCA |
| LmjF35.4930 | CATGCGTTGTCGTGGAAGAGA | TTTCGACGGAAGAGCACGTG |
| LmjF36.0070 | CATGCAGGTAGCGAAACGTCA | CTGCGCACTGTCTACATCAC |
| LmjF36.1250 | GGCTTCCTCGACGAGGAGAA | CTCGAGAATGTCCGGCACC |
| LmjF36.3760 | GTCACGATGGCGATCAACTTC | TGATGTACGCCGACTTCAGGT |
| LmjF36.5880 | GGGAGCACGGGAGGTGTAA | CTGCACGCGCCATGAAC |
| rRNA45Lm | CCTACCATGCCGTGTCCTTCTA | AACGACCCCTGCAGCAATAC |
